# C-851-08. Artificial Intelligence-Enhanced Multispectral Imaging for Burn Wound Assessment: Pilot Evaluation of Complex Anatomical Sites

**DOI:** 10.1093/jbcr/irag033.060

**Published:** 2026-04-06

**Authors:** Shreyas V Supparamaniam, Poh (Leslie) Tan, Christopher J Lewis

**Affiliations:** Pinderfields General Hospital, Wakefield, England, Newcastle, England; Royal Victoria Infirmary, Newcastle, Newcastle upon Tyne, England; Northern Regional Burn Centre, Newcastle Upon Tyne, England

## Abstract

**Introduction:**

Burn depth assessment remains challenging, particularly in anatomically complex regions. Artificial intelligence (AI)-enhanced multispectral imaging (MSI) has demonstrated strong diagnostic accuracy in validated anatomical sites such as the trunk and limbs. However, its performance in sites such as hands, feet, face, and joints has not been validated. This pilot evaluation assesses the system’s application in these regions.

**Methods:**

A single-centre product evaluation was conducted at our Regional Burns Centre. Adults (≥18 years) with superficial to full-thickness burns of the hands, feet, face, or joints were included. Exclusion criteria were chemical burns and patients undergoing surgery. AI-generated predictions were compared to healing assessment at 21 days and analysed using ImageJ, and statistical analyses were performed in JASP. Pixel-level pooling was used to calculate diagnostic performance, with Wilson 95% confidence intervals.

**Results:**

The evaluation included 33 patients and 37 burn images, representing approximately 2.4-million-pixel data points. Pixel-level pooled analysis in complex sites demonstrated a sensitivity of 79.0% (95% CI 78.8–79.3%), a specificity of 91.4% (95% CI 91.4–91.4%), and an overall accuracy of 90.8% (95% CI 90.8–90.9%). The positive predictive value was 31.4% (95% CI 31.2–31.5%), while the negative predictive value remained high at 98.9% (95% CI 98.9–98.9%).

**Conclusions:**

Compared with validated anatomical sites, diagnostic performance in complex regions was lower for specificity and PPV but maintained high NPV. These findings suggest potential clinical utility in complex regions such as the hands, feet, face, and joints, pending formal manufacturer validation.

**Applicability of Research to Practice:**

This study provides early evidence that AI-enhanced MSI may extend its utility to burns in anatomically complex regions, supporting clinical decision-making where assessment is most difficult. Although diagnostic performance was lower than in previously validated sites, the high NPV indicates a strong role in ruling out deep injury, potentially reducing unnecessary surgical intervention. Translation into practice will require larger multicentre validation studies, comparison with current standards such as laser Doppler imaging, and assessment of workflow integration and usability in real-time clinical settings. Nonetheless, this work highlights the technology’s potential to improve reproducibility and confidence in burn depth assessment in complex areas, marking a technological progression.

**Funding for the study:**

N/A.

